# Digital versus conventional impression accuracy for complete-arch implant rehabilitation: An *in vitro* study

**DOI:** 10.6026/973206300221854

**Published:** 2026-03-31

**Authors:** Krishnendhu Sai, Byju Paul Kurian, Litto Manual, Shalu Sara Aby, Sandra S Anand, Fathima Mohamad Najeeb, Vrinda Nandakumar

**Affiliations:** 1 Department of Prosthodontics, Mar Baselios Dental College, Kothamangalam, Kerala, India

**Keywords:** Digital impression, conventional impression, implants, accuracy

## Abstract

Accurate implant position transfer remains critical for passive fit in full-arch implant prostheses, particularly challenging with angulated implants in edentulous mandibles. This *in vitro* study compared ten digital impressions (TRIOS scanner) versus ten conventional splinted open-tray polyether impressions using an edentulous mandibular model with six implants. Three-dimensional deviations were measured via coordinate measuring machine, revealing no statistically significant differences between techniques via independent t-test analysis. Conventional impressions demonstrated slightly superior X- and Y-axis precision, while digital impressions shows marginally better Z-axis trueness, both proves clinically acceptable. These findings advance prosthodontic practice by validating both digital and conventional impression techniques for complete-arch implant rehabilitation while highlighting conventional method predictability for complex cases.

## Background:

Precise impression making is a critical step in implant prosthodontics, particularly in complete arch rehabilitation, where multiple implants must be accurately transferred to achieve passive fit of the definitive prosthesis [1]. Misfit of implant-supported prostheses has been associated with mechanical complications such as screw loosening, component fracture and biological sequelae, including peri-implant bone loss and soft tissue inflammation [2]. Conventional implant impression techniques, particularly the splinted open tray method using polyether or polyvinyl siloxane materials, have long been considered the gold standard for multi-implant restorations due to their proven dimensional stability and accuracy [3]. However, these techniques are technique-sensitive, time-consuming and may cause patient discomfort, especially in full arch situations [4]. The introduction of digital intraoral scanners has transformed prosthodontic workflows by permitting direct acquisition of three-dimensional implant position data and eliminating the need for conventional impression materials. Research reported that digital impressions enhance patient comfort, shorten chairside time and integrate CAD/CAM systems efficiently [5]. However, limitations are still reported, including potential stitching errors, scan body design, implant angulation and reduced accuracy in edentulous arches with multiple implants [6]. Several contemporary investigations have demonstrated that digital impressions can achieve accuracy comparable to conventional methods in partial edentulism, while evidence for complete arch implant rehabilitation remains inconclusive [7, 8]. Therefore, is of interest to further evaluate digital versus conventional impression techniques under controlled conditions is essential to guide evidence-based clinical decision making.

## Methodology:

This *in vitro* comparative study was conducted at the Department of Prosthodontics, Mar Baselios Dental College, Kothamangalam, Kerala, in collaboration with Amrita Engineering College, Kollam. An *in vitro* experimental study was conducted to compare the accuracy of digital and conventional impression techniques for complete-arch implant rehabilitation. A standardised edentulous mandibular master model was fabricated to simulate clinical conditions. Molten modelling wax was poured into a prefabricated edentulous silicone mould to obtain a preliminary wax pattern. The residual ridge was standardised to a height of 13 mm and a width of 7 mm. This wax pattern was replicated using silicone and a definitive master model was fabricated using heat-cured clear acrylic resin. A comprehensive digital treatment plan was developed and based on this plan; a customised Osstem surgical guide was fabricated using CAD/CAM technology. The guide incorporated predetermined channels to control implant angulation and depth during placement. Using the Osstem guided surgery kit, six implants were placed in the acrylic master model with the aid of anchoring pins to stabilise the guide (Figure 1 - see PDF). Four straight implants (4 x 11.5 mm) were positioned in the anterior region, while two posterior implants (4 x 13 mm) were placed in the premolar regions with approximately 30°C mesial angulation to simulate tilted implant placement commonly used in full-arch rehabilitation. Multiunit abutments were connected to all implants to standardise the restorative platform. Ten custom open trays were fabricated using light-cure tray material. A 4-mm wax spacer with stops was adapted over the master model, tray material was polymerised using a UV curing unit and openings were created to accommodate open-tray transfer copings and adapted with wax for supporting impression material (Figure 2 - see PDF). The samples were divided into two groups (n = 10 each). In Group A (digital impression group), scan bodies were attached to the implants and the master model was coated with a cement spacer to minimise reflection from the transparent acrylic surface. Digital impressions were obtained using a TRIOS 3Shape intraoral scanner. The scans were exported as stereolithography (STL) files and used to fabricate ten 3D-printed models using a Formlabs Form 4 printer. In Group B (conventional impression group), open-tray transfer copings were secured to the multiunit abutments with a torque of 15 Ncm. The copings were splinted using dental floss and pattern resin (Figure 3 - see PDF), allowed to polymerise and impressions were made using polyether impression material with an automatic mixing unit (Figure 4 - see PDF). After loosening the coping screws, the impressions were removed and sent for cast fabrication. Study casts were produced by attaching implant analogues to the transfer copings and pouring type IV dental stone under standardised water-powder ratios and vibration. All casts were allowed to set before separation. Titanium cylinders were attached to the implant analogues on the master model (Figure 5 - see PDF), digital models and conventional casts to facilitate precise and reproducible measurements. A coordinate measuring machine (CMM) was used to record three-dimensional coordinates (x, y and z axes) of the master model and all test models (Figure 6 - see PDF). Implants were sequentially numbered from left premolar to right premolar region to standardise measurements. Inter-implant distances between implant 1 and implants 2 through 6 were calculated and linear deviations in x, y and z axes, as well as angular deviations along the z-axis, were recorded. The three-dimensional discrepancies of both digital and conventional impression groups were compared with the master acrylic reference model to evaluate the accuracy of each impression technique.

## Results:

Significant differences in implant position accuracy were observed between the two groups. In the Z-axis inter-implant distance analysis, Group A (digital impressions) showed statistically significant deviations from the master model in selected measurements (Differences 1, 3 and 4; p < 0.05), while Group B (conventional impressions) demonstrated minimal and largely non-significant deviations. Angulation analysis revealed significant discrepancies in Group A for Angulations 3 and 4 (p < 0.05), whereas Group B showed no significant differences except for Angulation 5 (p < 0.001) (Table 1 - see PDF). All measured parameters showed similar accuracy for both digital and conventional impression techniques. Statistical analysis showed no significant difference between the two groups for inter-implant distances, linear coordinate measurement along the X, Y and Z axes, or angular deviations (p > 0.05). Descriptive observation, however, shows slightly improved horizontal accuracy (X and Y axes) with the conventional splinted open-tray method, particularly in areas with angulated implants (Table 2 - see PDF). In contrast, the digital workflow exhibited marginally improved vertical accuracy (Z axis) and consistency in certain inter-implant distance measurements (Table 3 - see PDF). Despite these minor variations, the magnitude of deviations in both groups remained within clinically acceptable limits, confirming that each method enabled reliable transfer of implant positions in full-arch cases.

## Discussion:

The accuracy of implant impression techniques plays a critical role in achieving passive fit and biomechanical stability of complete arch implant-supported prostheses. The present study demonstrated that both digital and conventional impression techniques show clinically acceptable accuracy. Conventional splinted open tray impressions method showed marginally reduced horizontal deviations in the X and Y axes when compared with digital impressions. This finding supports that the rigid splinting of impression copings minimises positional movement during impression removal. The study indicates that both digital and conventional impression techniques can achieve clinically acceptable accuracy for complete arch implant rehabilitation. This aligns with recent systematic reviews reporting comparable trueness between intraoral scanning and conventional impressions for full arch implant restorations [9, 10]. Conventional splinted open tray impressions showed slightly superior precision when implants were angulated, which may be attributed to the rigid stabilisation of impression copings and minimises positional distortion during impression retrieval [11]. Similar observations were reported by Park *et al.* who noted that conventional impressions remain more predictable in complex multi-implant cases [12]. Digital impressions, on the other hand, provide notable benefits in terms of patient comfort, reduced working time and more efficient digital workflows. Suhael *et al.* stated that patients consistently preferred digital impressions compared with conventional techniques due to reduced discomfort and gag reflex [13]. While these advantages favour digital workflows, clinicians must weigh them against the potential limitations observed in complex implant scenarios. This study has certain limitations. The *in vitro* design, which could not, simulates intraoral conditions such as saliva, soft tissue movement and limited intraoral scanning access. Furthermore, the evaluation was limited to a single intraoral scanner. Future *in vivo* studies incorporating multiple scanner systems and clinical variables are necessary to validate these findings. Recent studies have highlighted that improvements in scanner technology, scan body design and scanning protocols markedly improved the accuracy of digital impressions even in edentulous arches [14]. However, factors such as implant angulation, inter- implant distance and cumulative stitching errors can still influence accuracy in full arch cases [15]. Overall, the present results support the expanding evidence that digital impressions are a reliable alternative to conventional methods, while highlighting the continued relevance of splinted open tray techniques in situations demanding maximum precision.

## Conclusion:

Both digital and conventional implant impression techniques were capable of producing clinically acceptable accuracy for full-arch restorations, although variation in dimensional stability was evident. Conventional splinted open-tray polyether impressions showed the closest value with the master model implant positions, indicating greater reliability, particularly in cases with multiple or angulated implants where passive fit is critical. Digital impressions remain a useful alternative in less complex situations, but further clinical research is needed to optimise their use in full-arch implant prosthodontics.

## References

[1] Richi MW (2020). Head Face Med..

[2] Verma A (2023). J Oral Biol Craniofac Res..

[3] Rajendran R (2021). Eur Oral Res..

[4] Froimovici FO (2024). Dent J (Basel)..

[5] Schmidt A (2021). Clin Oral Implants Res..

[6] Marques S (2021). Int J Environ Res Public Health..

[7] Drancourt N (2023). J Pers Med..

[8] Albanchez-González MI (2022). Int J Environ Res Public Health..

[9] Kernen-Gintaute A (2025). J Dent..

[10] Pesce P (2024). J Clin Med..

[11] Patil P (2023). BMC Oral Health..

[12] Park JS (2025). J Dent..

[13] Ahmed S (2024). Cureus..

[14] Achmadi AA (2025). BDJ Open..

[15] Muslu T, Kurtulmus-Yilmaz S (2025). BMC Oral Health..

